# Orthopedic Injuries in Beach Tennis Players in Brazil

**DOI:** 10.1055/s-0044-1786521

**Published:** 2024-06-22

**Authors:** Antonio Carlos da Costa, Luana Baptistele Dornelas, Marina Rafaele Makishi

**Affiliations:** 1 Grupo de Cirurgia da Mão e Microcirurgia, Departamento de Ortopedia e Traumatologia, Santa Casa de São Paulo, São Paulo, SP, Brasil; 2 Faculdade de Ciências Médicas, Santa Casa de São Paulo, São Paulo, SP, Brasil

**Keywords:** beach tennis, epidemiology, sports medicine, tennis/injuries

## Abstract

**Objective** To analyze the epidemiological data of orthopedic injuries in beach tennis players.

**Methods** We conducted a cross-sectional study, through a questionnaire, with 185 beach tennis players during tournaments and training sessions in two cities. The questionnaire comprised anthropometric data, as well as data on length of time practicing the sport and reported orthopedic injuries.

**Results** We excluded 25 of the 185 interviewees. Of the 160 players studied, 51.9% were male and 48.1%, female. The average age was of 40.4 years, the average height was of 1.73 m, the average weight was of 75.6 kg, and the average body mass index (BMI) was of 25.2 kg/m ^2^ . Orthopedic injuries were reported by 48.8% of the participants, 30.0% in the lower limbs, 11.3% in the spine, and 25.0% in the upper limbs, with an incidence of 0.82 per one thousand hours of practice. Correlating the incidence of injuries with age, weight, height, and BMI, we only found relevance between the higher average age in patients with lower- and upper-limb injuries. Players who had no previous experience with other racket sports had fewer injuries. Other parameters, such as gender, use of the two-handed *backhand* , dominant side, participation in competitions, and practice of other sports did not show statistically significant differences.

**Conclusion** Orthopedic injuries were found in almost half of the beach tennis players, primarily in the lower limbs. Age, experience with other racket sports, category, hours of training per week, and length of time playing the sport influenced the incidence of orthopedic injuries.

## Introduction

Beach tennis (BT) was created in Italy around 1987, 1 and it arrived in Brazil in 2008. 2 The Brazilian Beach Tennis Confederation estimates around three hundred thousand players, with a significant growth following the pandemic. 2 The sport is a mixture of traditional tennis, beach volleyball, and badminton. 3

Despite the considerable number of players, we have found only one article 1 on orthopedic injuries in BT, from a study performed in France. There have been several studies on injuries in field tennis players, but these data cannot be transposed to BT since court and sand sports differ not only in the surface but also in the racket and the kinematics of the sport. 3

The present study aims to obtain epidemiological data on orthopedic injuries in BT players, to enable health professionals to get to know possible injuries and establish treatment and prevention targets for Brazilian players.

## Materials and Methods

A cross-sectional study was conducted through a questionnaire applied in person by the same author to 185 BT players during tournaments and training sessions at clubs in the cities of São Paulo and São Caetano do Sul, Brazil, between September 2021 and January 2022. All players agreed to participate voluntarily in the interview. The inclusion criteria were individuals over 18 years of age who had been practicing the sport regularly for at least 6 months. Interviewees who were unable to complete the questionnaire were excluded.

In the first part, the following data was collected: age, gender, height, weight, body mass index (BMI), and dominant side. In the second part, we investigated practice time (in months, days per week, and hours per week), participation in competitions, category, experience with other sports, and use of the backhand with both hands. The classification by category was self-reported and divided, according to performance in competitions, into “Pro” (professional), followed by categories A, B, C, and beginner.

The last part was the collection of data related to the presence of injuries and the segment in which they had occurred: the spine, upper limbs (ULs), and lower limbs (LLs). The injuries were self-reported.

Regarding the analysis of injuries, previous orthopedic injuries that could interfere with the practice of BT were disregarded. If an interviewee reported a previous asymptomatic injury, this was not considered when analyzing the data .

To obtain the incidence of injuries for every one thousand hours, the time spent practicing the sport for each participant was determined by multiplying the variables “training time (hours/week)” and “time spent practicing (months),” considering an estimate of four weeks in each month.

The data was analyzed using the Student *t* -test, the Chi-squared test, and the test of equality of two proportions, using the software IBM SPSS Statistics for Windows (IBM Corp., Armonk, NY, United States), version 20.0, Minitab 16 (Minitab, LLC, State College, PA, United States), and Excel Office 2010 (Microsoft Corp., Redmond, WA, United States), and considering a significance level of 5%.

The present study was approved by the Brazilian National Research Ethics Committee under CAAE 56741122.8.0000.5479.

## Results

Of the 185 players, 25 were excluded due to questionnaires that had not been fully filled out. Of the 160 practitioners analyzed, 83 were male (51.9%) and 77 were female (48.1%). The average age was of 40.4 years, ranging from 18 to 66 years. The average height was of 1.73 m, the average weight was of 75.6 kg, and the average BMI was of 25.2 kg/m ^2^ .

Of those interviewed, 144 were right-handed, and 16 were left-handed. Concerning the length of time they had been practicing the sport, most (46.3%) had been practicing for up to 12 months, followed by 33.8% who had been practicing between 13 and 36 months, and 20%, over 36 months. Most players trained for 2 to 4 hours per week, accounting for 28.1%, and only 16.3% trained for more than 10 hours per week. In total, 71 (44.4%) players were beginners, 34 (21.3%) were in category C, 40 (25.0%) were in category B, and only 15 (9.4%) were in categories A or Pro. Many had practiced racket sports before (56.9%), and 52.5% did not practice other sports concurrently.

The presence of orthopedic injuries was reported by 78 practitioners (48.8%). The highest prevalence was found in the LLs, accounting for 48 injuries (30.0% of all injuries). Regarding the other segments, 18 spinal injuries (11.3%) and 40 UL injuries (25.0%) were reported.

A total of 94,572 hours of practicing the sport were obtained, with 78 injuries, with an incidence of 0.82 injuries per one thousand hours practiced (95% confidence interval [95%CI]: 0.18).

Concerning spinal injuries, 55.6% of the participants had spinal disc problems. In the ULs, the injuries with the highest prevalence were tendinopathy (47.5%) and epicondylitis (32.5%). In the LLs, knee injuries were the most common, with meniscus injuries (31.3%), followed by patellofemoral pain (25%), and ligament injuries (14.6%).

Correlating the incidence of injuries with age, weight, height, and BMI, there was only a statistically significant difference between the mean age of individuals with LL injuries (44.3 years with injury versus 38.8 years without injury) and UL injuries (45.6 years with injury versus 38.7 years without injury), with no statistical difference in spinal injuries.

We also found that players with no previous experience in other racket sports had fewer injuries (52.4%; *p* = 0.015). In addition, the prevalence of injuries found according to the length of time the players had been practicing BT was not proportional. In players who had been practicing for up to 12 months and in those practicing with more than 36 months of practice, 28.2% and 25.6% of injuries were found respectively; thus, the highest number of players with orthopedic injuries was found among those who had been practicing between 13 and 36 months (33.8%; *p* < 0.001) ( Table 1 ). There was a higher incidence of injury in individuals who trained more hours per week ( *p* = 0.007) ( Table 1 ).

**Table 1:** Correlation between players with and without injury and the parameters “experience in other racket sports,” “time spent practicing BT,” “category,” and “BT training time”

		Without injury		With injury		Total		*p* -value
		N	%	N	%	N	%	
Experience in other racket sports	No	43	52.4%	26	33.3%	69	43.1%	0.015
	Yes	39	47.6%	52	66.7%	91	56.9%	
Time spent practicing BT (months)	Up to 12	52	63.4%	22	28.2%	74	46.3%	< 0.001
	From 13 to 36	18	22.0%	36	46.2%	54	33.8%	
	More than 36	12	14.6%	20	25.6%	32	20.0%	
Category	A/Pro	6	7.3%	9	11.5%	15	9.4%	< 0.001
	B	10	12.2%	30	38.5%	40	25.0%	
	C	12	14.6%	22	28.2%	34	21.3%	
	Beginner	54	65.9%	17	21.8%	71	44.4%	
BT training time (hours/week)	0–2 hours	26	31.7%	13	16.7%	39	24.4%	0.007
	2–4 hours	24	29.3%	21	26.9%	45	28.1%	
	4–6 hours	14	17.1%	8	10.3%	22	13.8%	
	6–8 hours	5	6.1%	6	7.7%	11	6.9%	
	8–10 hours	8	9.8%	9	11.5%	17	10.6%	
	> 10 hours	5	6.1%	21	26.9%	26	16.3%	

**Abbreviations:** BT, beach tennis; Pro, professional.

The time spent practicing BT and the player category were statistically relevant regarding the presence of injuries. In the spine, players who had been practicing between 13 and 36 months were found to have 50% of injuries, while in the ULs, the rate was of 45%, and, for the LLs, it was of 47.9% for the same length of time practicing the sport. In category B players, around 33.3% had spinal injuries, 37.5% had UL injuries, and 39.6% reported LL injuries. Other parameters, such as gender, two-handed backhand, dominant side, participation in competitions, and practice of other sports did not show statistically significant differences.

## Discussion

The present study provides information on BT injuries in the Brazilian population. Although the total number of participants was relatively small and concentrated in a single metropolitan region, the study showed a homogeneous population, with no statistical differences in terms of gender, age, weight, or height of the players. In the present study, orthopedic injuries were considered all symptomatic injuries reported by the athlete that arose or worsened due to practicing BT. 4

As for the incidence of injuries, 78 (48.8%) players had orthopedic injuries, a figure similar to that found in the study by Berardi et al. 1 We obtained 0.82 injuries for every one thousand hours played, a figure slightly different from that of the survey by Berardi et al. 1 , with an incidence of 1.81 injuries, and from studies on tennis, 5 6 7 8 with an incidence of 1.5 to 20 injuries for every one thousand hours played. We believe that because we interviewed active players, and most of them during competitions, injured players may have been left out of the study.

Injuries occurred mainly in the LLS, followed by the ULs and the spine. This distribution of locations differed slightly from that described by Berardi et al., 1 with the most injuries occurring in the ULs (48.3%), followed by the LLs (43.4%), and the torso (8.4%). However, reviews of epidemiological studies on tennis show a distribution of injuries similar to that found in the present study, with LL injuries being the most common (31% to 67%), followed by the ULs (20% to 49%), and the torso/spine (3% to 21%).

The shoulder was the most injured site in the ULs in the French 1 and Brazilian analyses. In contrast, studies on tennis players 5 6 7 8 found that the elbow was most affected, with lateral epicondylitis being the most prevalent. This difference between tennis and BT is probably due to the biomechanics of each sport. In BT, most movements occur above the head, which probably leads to more shoulder injuries. 1

In the LLs, injuries occurred more in the knee. However, in the previous study on BT, 1 the most common injuries were to the thigh and feet. The ankle and thigh are the most common injury sites in tennis players. 5 It is recognized that sand sports require more energy expenditure and muscle work when compared to hard court sports; 9 10 therefore, the ankle, hip, and knee may be more injured. However, this data was not found in the study by Berardi et al., 1 but corroborated the data found in the present study, with higher rates of injuries reported in the LLs.

In the present study, patients with a reported orthopedic injury had a higher average age when compared to those without injuries ( *p* < 0.001); this was also verified regarding the location of the injury, which remained statistically relevant in UL and LL injuries, but not significant for spinal injuries. The same was not found in the other studies on BT 1 and tennis. 5 Thoroughly analyzing the studies, the average age in the present study is higher than in the others. This may have made age a relevant factor in the present analysis.

A higher injury rate was found in those who had already practiced or were practicing another similar sport (66.7%). In addition, the length of time practicing the sport also seemed to influence the appearance of injuries, as did the category of the players. Practicing BT for up to 12 months and spending less time training per week resulted in lower rates of orthopedic injuries, while the beginner category also had fewer reported injuries. In previous studies on tennis, 5 it is recognized that a greater volume of training or playing is correlated with the presence of injuries; however, the beginner category of players, be them of tennis 5 or BT, 1 seems to present a higher rate of injuries, which differs from the data found in the present study.

Other parameters, such as gender, dominant side, use of the backhand with both hands, participation in competitions, and practice of other sports were not statistically significant in players with or without orthopedic injuries in the present study.

The limitations of the present study were mainly found in the cross-sectional design, in which there was no differentiation between chronic and acute injuries. In addition, the diagnosis of the orthopedic injury was based on the athlete's report, which can lead to bias in data collection.

## Conclusion

Orthopedic injuries were found in almost half of the BT players, preferably in the LLs, with an incidence of 0.82 injuries for every one thousand hours played. Age, experience with other racket sports, category, hours of training per week, and length of time practicing the sport influenced the incidence of orthopedic injuries.
