# Supplementary material for: Orthopedic Injuries in Beach Tennis Players in Brazil
Source: Rev Bras Ortop (Sao Paulo). 2024 Jun 22;59(3):e415–9. doi: 10.1055/s-0044-1786521 (PMC11193591; doi:10.1055/s-0044-1786521)
Supplement: Related texts

# Research article: Lesões ortopédicas nos praticantes de *beach tennis* no Brasil

**Keywords:** beach tennis, epidemiologia, medicina esportiva, tênis/lesões

## Abstract

**Objetivo** Analisar os dados epidemiolügicos das lesões ortopédicas nos praticantes de *beach tennis* .

**Métodos** Foi realizado um estudo transversal, por meio de questionário, em 185 praticantes de *beach tennis* , durante torneios e treinos, em duas cidades. O questionário consistiu na coleta de dados antropométricos, além de dados relativos ao tempo de prática do esporte e às lesões ortopédicas referidas.

**Resultados** Excluímos 25 dos 185 entrevistados. Dos 160 praticantes estudados, 51,9% eram do sexo masculino, e 48,1%, do feminino. A média de idade foi de 40,4 anos, a média da estatura foi de 1,73 m, a média do peso, de 75,6 kg, e a média do índice de massa corporal (IMC), de 25,2 kg/m ^2^ . A presença de lesões ortopédicas foi relatada por 48,8% dos praticantes, sendo 30,0% nos membros inferiores (MMII), 11,3% na coluna, e 25,0% nos membros superiores (MMSS), com incidência de 0,82 a cada mil horas de prática. Correlacionando a incidência de lesões com idade, peso, altura e IMC, encontramos relevância apenas entre a média de idade maior nos pacientes com lesão nos MMII e MMSS. Os jogadores que não tinham experiência prévia com outros esportes com raquete tiveram menos lesões. Demais parâmetros, como sexo, uso do *backhand* com duas mãos, lado dominante, participação em competições e prática de outros esportes não obtiveram diferenças estatisticamente significantes.

**Conclusão** Lesões ortopédicas foram encontradas em quase a metade dos praticantes de *beach tennis* , preferencialmente nos MMII. Idade, experiência com outros esportes de raquete, categoria, horas de treino por semana e tempo de prática do esporte influenciaram na incidência de lesões ortopédicas.

## Introdução

O *beach tennis* (BT) foi criado na Itália em meados de 1987 1 e chegou ao Brasil em 2008. 2 A Confederação Brasileira de Beach Tennis estima que haja cerca de trezentos mil praticantes, com grande crescimento apüs a pandemia. 2 O esporte é uma mistura do tênis tradicional, vôlei de praia e *badminton* . 3

Apesar do considerável número de praticantes, até a data do estudo encontramos apenas um artigo 1 sobre as lesões ortopédicas na prática do BT, de um estudo realizado na França. Existem diversos estudos sobre as lesões nos praticantes de tênis de campo; contudo, esses dados não podem ser transpostos para o BT, visto que os esportes de quadra e de areia diferem não sü na superfície, como também na raquete e na cinemática do esporte. 3

O objetivo deste estudo é obter dados epidemiolügicos das lesões ortopédicas nos praticantes de BT, para permitir que os profissionais da saúde conheçam as possíveis lesões, e para estabelecer metas de tratamento e prevenção nos praticantes brasileiros.

## Materiais e Métodos

Foi realizado um estudo transversal, por meio de questionário aplicado de maneira presencial pela mesma autora, em 185 praticantes de BT, durante torneios e treinos em clubes das cidades de São Paulo e São Caetano do Sul, Brasil, entre setembro de 2021 e janeiro de 2022. Todos concordaram em participar voluntariamente da entrevista. Como critérios de inclusão foram considerados indivíduos acima de 18 anos de idade, e praticantes do esporte regularmente por pelo menos 6 meses. Foram excluídos os entrevistados que não puderam responder ao questionário completo.

Na primeira parte, foram coletados os seguintes dados: idade, sexo, altura, peso, índice de massa corporal (IMC) e lado dominante. Na segunda parte, investigamos o tempo de prática (em meses, dias por semana e horas por semana), a participação em competições, a categoria, a experiência com outros esportes e uso de *backhand* com as duas mãos. A classificação por categorias foi autorrelatada e é dividida, de acordo com o desempenho em competições, em “Pro” (profissional) seguida das categorias A, B, C e iniciante.

A última parte foi a coleta de dados relacionadas à presença de lesões, e em qual segmento elas se apresentavam: coluna, membros superiores (MMSS) e membros inferiores (MMII). As lesões foram autorreferidas.

Com relação à análise das lesões, foram desconsideradas as lesões ortopédicas prévias que pudessem interferir na prática de BT. Caso um entrevistado relatasse lesão prévia assintomática, ela não foi considerada na análise dos dados do presente estudo.

Para obter a incidência de lesões a cada mil horas, foi determinado o tempo de prática do esporte de cada participante, multiplicando as variáveis “tempo de treino (horas/semana)” e “tempo de prática (meses)”, considerando a estimativa de quatro semanas a cada mês.

Os dados foram analisados por meio dos testes *t* de Student, do qui-quadrado, e de igualdade de duas proporções, utilizando os programas IBM SPSS Statistics for Windows (IBM Corp., Armonk, NY, Estados Unidos), versão 20.0, Minitab 16 (Minitab, LLC, State College, PA, Estados Unidos) e Excel Office 2010 (Microsoft Corp., Redmond, WA, Estados Unidos), e considerando o nível de significância de 5%.

Este estudo foi aprovado pelo Comissão Nacional de Ética em Pesquisa sob o CAAE 56741122.8.0000.5479.

## Resultados

Dos 185 praticantes, 25 foram excluídos devido a preenchimento incompleto do questionário. Dos 160 praticantes analisados, 83 eram do sexo masculino (51,9%), e 77, do feminino (48,1%). A média de idade foi de 40,4 anos, com variação de 18 a 66 anos. A média da estatura foi de 1,73 m, a média do peso, de 75,6 kg, e a média do IMC, de 25,2 kg/m ^2^ .

Entre os entrevistados, 144 eram destros e 16, canhotos. Com relação ao tempo da prática esportiva, a maioria (46,3%) dos entrevistados tinha até 12 meses, seguidos de 33,8% que tinham entre 13 e 36 meses, e 20% com mais de 36 meses. A maioria dos jogadores (28,1%) treinava por 2 a 4 horas semanais, e somente 16,3% treinava mais de 10 horas por semana. Ao todo, 71 (44,4%) praticantes eram iniciantes, 34 (21,3%) eram da categoria C, 40 (25,0%), da B, e apenas 15 (9,4%) eram das categorias A ou Pro. Muitos deles já praticavam esportes com raquete anteriormente (56,9%), e 52,5% não praticavam outros esportes concomitantes.

A presença de lesões ortopédicas foi relatada por 78 praticantes (48,8%). A maior prevalência encontrada foi nos MMII, com 48 lesões (30,0% de todas lesões). Quanto aos outros segmentos, foram relatadas 18 lesões na coluna (11,3%), e 40 lesões nos MMSS (25,0%).

Obteve-se o total de 94.572 horas de prática do esporte, com 78 lesões, o que significa uma incidência de 0,82 lesões a cada mil horas praticadas (intervalo de confiança de 95% [IC95%]: 0,18).

Em relação às lesões na coluna, foram encontradas afecções do disco vertebral em 55,6% dos praticantes. Já nos MMSS, as maiores prevalências de lesões foram as tendinopatias (47,5%) e epicondilite (32,5%). Nos MMII, as lesões de joelho foram as mais comuns, com lesão do menisco (31,3%), seguida de dor patelofemoral (25%), e lesão ligamentar (14,6%).

Correlacionando a incidência de lesões com idade, peso, altura e IMC, obteve-se relevância estatística significante apenas entre a média de idade maior nos indivíduos com lesão nos MMII (44,3 anos com lesão *versus* 38,8 anos sem lesão), e nos MMSS (45,6 anos com lesão *versus* 38,7 anos sem lesão), sem diferença estatística nas lesões de coluna.

Também verificou-se que os jogadores que não tinham experiência prévia com outros esportes com raquete tiveram menos lesões (52,4%; *p* = 0,015). Além disso, a prevalência encontrada de lesões conforme o tempo de prática do BT não foi proporcional. Nos praticantes até 12 meses e naqueles com mais de 36 meses, foram encontradas 28,2% e 25,6% de lesões, respectivamente; assim, o maior número de jogadores com lesões ortopédicas foi encontrado nos praticantes entre 13 e 36 meses (33,8%; *p* < 0,001) ( Tabela 1 ). Houve maior incidência de lesão em indivíduos que treinavam mais horas por semana ( *p* = 0,007) ( Tabela 1 ).

**Tabela 1:** Correlação entre praticantes com e sem lesão e os parâmetros “experiência em outros esportes com raquete,” “tempo de prática de BT,” “categoria” e “tempo de treino de BT”

		Sem lesão		Com lesão		Total		*p*
		N	%	N	%	N	%	
Experiência em outros esportes com raquete	Não	43	52,4%	26	33,3%	69	43,1%	0,015
	Sim	39	47,6%	52	66,7%	91	56,9%	
Tempo de prática de BT (meses)	Até 12	52	63,4%	22	28,2%	74	46,3%	< 0,001
	De 13 a 36	18	22,0%	36	46,2%	54	33,8%	
	Mais de 36	12	14,6%	20	25,6%	32	20,0%	
Categoria	A/Pro	6	7,3%	9	11,5%	15	9,4%	< 0,001
	B	10	12,2%	30	38,5%	40	25,0%	
	C	12	14,6%	22	28,2%	34	21,3%	
	Iniciante	54	65,9%	17	21,8%	71	44,4%	
Tempo de treino de BT (horas/semana)	0–2 horas	26	31,7%	13	16,7%	39	24,4%	0,007
	2–4 horas	24	29,3%	21	26,9%	45	28,1%	
	4–6 horas	14	17,1%	8	10,3%	22	13,8%	
	6–8 horas	5	6,1%	6	7,7%	11	6,9%	
	8–10 horas	8	9,8%	9	11,5%	17	10,6%	
	> 10 horas	5	6,1%	21	26,9%	26	16,3%	

**Abreviaturas:** BT, *beach tennis* ; Pro, profissional.

O tempo de prática do BT e a categoria dos praticantes tiveram relevância estatística com relação à presença de lesão. Na coluna, nos jogadores com 13 a 36 meses de prática, a taxa de lesões foi de 50%, ao passo que nos MMSS foi de 45%, e nos MMII, de 47,9%, para o mesmo tempo de prática esportiva. Nos praticantes da categoria B, cerca de 33,3% tinham lesão na coluna, 37,5% tinham lesão nos MMSS, e 39,6% relataram lesão nos MMII. Demais parâmetros, como sexo, *backhand* com duas mãos, lado dominante, participação em competições e prática de outros esportes não obtiveram diferenças estatisticamente significantes.

## Discussão

O presente estudo apresenta dados relativos às lesões do BT na população brasileira. Apesar do número total de participantes ser relativamente pequeno e concentrado numa única região metropolitana, este trabalho demonstrou uma população homogênea, sem diferenças estatísticas em termos de sexo, idade, peso ou altura. Neste, estudo foram consideradas lesões ortopédicas todas as lesões sintomáticas relatadas pelo atleta que surgiram ou se agravaram devido à prática do BT. 4

Quanto à incidência das lesões, 78 (48,8%) praticantes tiveram lesões ortopédicas, dado semelhante ao encontrado no estudo de Berardi et al. 1 Obtivemos 0,82 lesões a cada mil horas praticadas, dado pouco diferente do apresentado no estudo de Berardi et al. 1 , com incidência de 1,81 lesões, e dos estudos sobre o tênis, 5 6 7 8 com incidência de 1,5 até 20 lesões a cada mil horas jogadas. Acreditamos que, pelo fato de entrevistarmos praticantes em atividade e, na maioria, durante competições, os praticantes lesionados possam ter ficado de fora do estudo.

As lesões ocorreram principalmente nos MMII, seguidos dos MMSS e da coluna. Essa distribuição das localizações diferiu discretamente daquela descrita no estudo de Berardi et al., 1 sendo maior nos MMSS (48,3%), seguidos dos MMII (43,4%), e do tronco (8,4%). Entretanto, nas revisões dos estudos epidemiolügicos sobre o tênis, as lesões seguiram uma distribuição semelhantes à deste trabalho, com as lesões dos MMII sendo as mais comuns (31% a 67%), seguidas das dos MMSS (20% a 49%) e, por último, das do tronco/coluna (3% a 21%).

Nos MMSS, o ombro foi o local mais lesionado, tanto na análise francesa 1 quanto na brasileira. Já nos trabalhos que avaliam os jogadores de tênis, 5 6 7 8 encontrou-se o maior acometimento do cotovelo, sendo a epicondilite lateral a mais prevalente. Essa diferença entre o tênis e o BT provavelmente deve-se à biomecânica de cada esporte. No BT, a maioria dos movimentos acontecem acima da cabeça, o que provavelmente leva a número maior de lesões no ombro. 1

Nos MMII, as lesões ocorreram mais no joelho. Contudo, no estudo prévio sobre o BT, 1 as lesões mais comuns se deram na coxa e nos pés. Nos praticantes de tênis, o tornozelo e a coxa são os locais mais comuns de lesão. 5 É reconhecido que os esportes na areia necessitam de mais gasto energético e trabalho muscular quando comparados aos esportes de quadra de superfícies duras; 9 10 portanto, tornozelo, quadril, e joelhos podem apresentar-se mais lesionados. Contudo, tais dados não foram encontrados no estudo de Berardi et al., 1 mas corroboram os dados encontrados no presente trabalho, com maior índice de lesões nos MMII.

Neste estudo, os pacientes que relataram ter lesão ortopédica tinham média de idade maior quando comparados com aqueles sem lesão ( *p* < 0,001); isso também foi verificado com relação à localidade da lesão, e permaneceu relevante estatisticamente nas lesões dos MMSS e MMII, mas sem relevância nas lesões da coluna. Nos demais estudos, tanto do BT 1 quanto do tênis, 5 o mesmo dado não foi encontrado. Avaliando em detalhes os estudos, pode-se verificar que a média de idade do presente trabalho é mais alta comparada às médias dos demais estudos. Tal fato pode ter feito da idade um fator relevante na presente análise.

Foi encontrado maior índice de lesão naqueles que já praticaram ou praticavam outro esporte similar (66,7%). Além do mais, o tempo de prática do esporte também pareceu influenciar o aparecimento de lesões, assim como a categoria dos praticantes. A prática do BT por até 12 meses e o menor tempo de treino por semana resultaram em índices menores de lesões ortopédicas, assim como também houve menos lesões relatadas entre a categoria iniciante. Nos estudos prévios sobre tênis, 5 é reconhecido que o maior volume de treino ou jogo tem correlação com a presença de lesão; contudo, a categoria iniciante dos praticantes tanto de tênis 5 quanto de BT 1 parece apresentar índice maior de lesões, o que difere dos dados encontrados no presente estudo.

Outros parâmetros, como sexo, lado dominante, uso do *backhand* com as duas mãos, participação em competições e prática de outros esportes não obtiveram relevância estatística nos praticantes com ou sem lesão ortopédica no presente estudo.

As limitações deste estudo se concentraram no seu desenho transversal, em que não foram diferenciadas lesões crônicas de lesões agudas. Além disso, o diagnüstico da lesão ortopédica foi baseado no relato do atleta, o que pode conferir viés na coleta dos dados.

## Conclusão

Foram encontradas lesões ortopédicas em quase a metade dos praticantes de BT, preferencialmente nos MMII, com incidência de lesões de 0,82 a cada mil horas praticadas. A idade, a experiência com outros esportes de raquete, a categoria, as horas semanais de treino e o tempo de prática do esporte influenciaram na incidência de lesões ortopédicas.
